# Leveraging Leadership in Child Welfare Systems: Large-scale Trauma- and Resilience-informed Training Initiative

**DOI:** 10.1007/s11414-022-09815-2

**Published:** 2022-08-25

**Authors:** Adriana Rodriguez, Zhe Fei, Wendy A. Barrera, Eugenia H. Tsao, Jill Waterman, Todd M. Franke, Catherine E. Mogil, Blanca Bonilla, Gita Murthy Cugley, Teri Gillams, Audra Langley

**Affiliations:** 1 University of California, Los Angeles, CA USA; 2 Jane and Terry Semel Institute for Neuroscience and Human Behavior, 760 Westwood Plaza, Los Angeles, CA 90095 USA; 3 UCLA Luskin School of Public Affairs, UCLA Pritzker Center for Strengthening Children and Families, Los Angles, CA USA; 4 Los Angeles County Department of Children and Families, Los Angeles, CA USA; 5 Los Angeles County Department of Mental Health, Los Angeles, CA USA; 6 University of California, UCLA Pritzker Center for Strengthening Children and Families, Los Angeles, CA USA; 7 Center for the Study of Healthcare Innovation, Implementation & Policy, VA Greater Los Angeles, Los Angeles, CA USA

## Abstract

Strengthening the infrastructure of public health systems around trauma-informed principles is crucial to addressing the needs of traumatized children in the child welfare system. In fact, many local and state initiatives have focused on large-scale evaluation studies to determine the value of training direct service staff on trauma foundations. Less yet is known about the benefits of training leaders on trauma foundations, which is crucial given their unique influence on implementation decisions. The current study evaluates a trauma training delivered to leadership-level stakeholders through a large-scale training initiative for the Los Angeles County Department of Children and Family Services. Findings indicated that leaders improved in trauma knowledge from baseline to post-training and reported changes in their professional wellbeing and leadership approach after the reflective training component. The leadership trauma program may have positive downstream implications for direct service staff, organizational culture, and child and family outcomes.

## Introduction

Children and youth in the child welfare system (CWS) experience high rates of childhood trauma, often worsened by stressors and trauma stemming from repeated separations and placement transitions.^1,2,3^ Many who enter the CWS have experienced multiple, chronic traumatic events perpetrated by a caregiver (child abuse and neglect), which typically is the precipitant for the separation from a caregiver.^4^ Although not all children exposed to trauma have symptoms of distress, an array of immediate and long-term consequences of trauma exposure is well-documented and multifaceted, including attachment impairments, increased risk for future trauma exposure, mental health challenges, emotion regulation difficulties, and poor self-concept.^5^ Awareness and education around trauma and its impacts on children can support efforts to combat these impacts. Strengthening the infrastructure of public health systems around trauma- and resilience-informed care principles is vital to addressing the needs of traumatized children involved in the CWS.

Numerous public health calls to action have emerged to prevent and ameliorate the impacts of trauma exposure on children broadly, and especially on children and youth involved in foster care.^6^ Efforts have focused on supporting and urging states to adopt trauma- and resilience-informed care practices and policies in systems of care, conceptualized as a system that understands the widespread impact of trauma, signs, and symptoms, and can respond by “…fully integrating knowledge about trauma into policies, procedures, and practices, and seeks to actively resist re-traumatization.”^6^ ^(p. 9)^ This push has given rise to evaluation studies of large-scale local and state initiatives focused on training service providers in trauma-focused evidence-based practices (EBPs).^7,8,9^ Little research has focused on evaluating the efforts to build capacity among leadership-level stakeholders, which is surprising given the infrastructural decision-making influence leaders have on organizations and teams in adopting and implementing wide-scale initiatives. Training for client interfacing staff, although key for supporting trauma- and resilience-informed systems of care, also requires support from leadership for organization-wide adoption and buy-in. The initial phase of a Los Angeles County, Department of Children and Family Services (DCFS) rollout of a trauma- and resilience- informed training program for DCFS leader stakeholders is described and evaluated in the current paper.

### Large-scale trauma- and resilience-informed initiatives in CWS

The evidence in support of trauma- and resilience-informed models within CWS is compelling. Several evaluation studies of statewide and local trauma- and resilience-informed care initiatives show improvements in staff trauma knowledge, attitude, and use of EBPs.^8–11^ The focus of these studies, however, has been on direct service mental health providers, as opposed to system leaders (e.g., executive leaders, managers, supervisors), which are often the first line for policy decision-making and key to successful implementation. The few studies that have focused on training leaders failed to adapt the curriculum to include leader-relevant content, often training on elements most appropriate for direct service providers (e.g., prevention, treatment, assessment).^8,9,11^ One study, for example, evaluated frontline staff and leader outcomes and found significantly greater knowledge gains among frontline staff compared to leaders, which authors speculate may suggest a need to adapt the training curriculum to the unique needs of each stakeholder. Trauma- and resilience-informed programs that forge relationships with system-level decision-makers prove key to implementation.^12^ The current study expands upon this research by evaluating the implementation of post-training leadership sessions (coined, Reflective Circles) for executive leaders, managers, and supervisors on providing an experiential opportunity using reflective practice and skills to implement trauma- and resilience-informed care and strategies within their organization.

### Guiding implementation framework

Child welfare systems represent a complex context that spans individual-, family, provider, organization, and system levels. Implementation science offers useful models and concepts that lend to the inquiry of large-scale rollout of trauma- and resilience-informed practices and policies for these systems. A frequently cited conceptual model of factors thought to be most critical for effective implementation of EBPs or initiatives in publicly funded child and family service is the four-phase EPIS framework (Exploration, Preparation, Implementation, Sustainment), which distinguishes inner from outer contexts that uniquely affect implementation in public service sectors (e.g., funding and system-wide policies vs. internal organization culture).^13^ The Exploration phase is focused on evaluating the needs and potential program or initiative fit; the Preparation phase is geared towards planning and outreach around the program once the decision has been made to adopt it; the Implementation phase, the focus of the current study, is the rollout of any program (in this case, the trauma- and resilience-informed training program for leadership across DCFS); and the Sustainment phase is focused on the continued use of the program in practice. The inner context issues around leadership engagement is the focus of the current study, through the ongoing training program and support for leaders. Although leadership alone is insufficient for effective implementation, leveraging leadership at all pertinent levels, combined with organizational supports, promotes a positive implementation climate, attitude, and readiness for change.^13,14^

### Role of leadership and its impacts on implementation

Executive directors, managers, and supervisors within CWS play a critical role in organizations due to their multitier leadership influence across levels of stakeholders. According to the Burke-Litwin Causal Model of Organization, a transformational leader, defined as someone who focuses on vision, change, influence, and provides opportunities to learn new skills, is crucial to organizational shift.^15^ Fostering and cultivating leaders that personally identify with the change needed within an organization (in this case, trauma- and resilience-informed care) serves as robust catalyst for change across the organization. Much of the research on determinants of effective implementation of policies and practices is consistent with this change model.^16^ The role of transformational leadership is also key to creating a trauma- and resilience-informed organizational culture.^15^ A recent study found that agency-level factors robustly explained the most variance in an organizations’ engagement with trauma- and resilience-informed practices, such that positive organizational culture was associated with the effective implementation of trauma- and resilience-informed organizations.^17^ The central role of effective leadership and organizational culture on trauma- and resilience-informed organizations is apparent, and therefore crucial to involve leaders in training to maximize the downstream impacts on child and family services in child welfare.

### Paper aims and hypotheses

The primary aim of the study was to evaluate the effectiveness of implementing a countywide trauma- and resilience-informed training program (1-day in-person training and ongoing 4-month Reflective Circles) for DCFS leadership. The primary outcomes were satisfaction, knowledge gain, and practice change. The relationship between knowledge and leader factors (education level, job role) were also evaluated, given the importance in past research of these individual-level variables in determining knowledge growth and knowledge.^11^ It was hypothesized that leaders with more education and in more administrative roles (i.e., less direct clinical care) will show substantial knowledge gains compared to those with less education and in more clinical care leadership roles. It was anticipated that leaders would report and describe themes of practice change across quantitative and qualitative data sources.

## Method

### Partnership-driven initiative context

In 2018, the Los Angeles County Department of Mental Health (LAC DMH) and the University of California, Los Angeles (UCLA) began a partnership to leverage the strengths of the two public institutions to strengthen communities, reengineer systems, and revitalize policy. The initiative focused on training and professional development for the LAC workforce across systems of care (e.g., mental health, education, child welfare). Training initiatives focused on systems where the county’s most vulnerable children receive services that aim to mitigate the impact of trauma and promote individual and community empowerment. Thus, the first year of the partnership focused on building capacity in the workforce across diverse sectors of LAC in trauma- and resilience-informed systems of care. The LAC DCFS, the largest child welfare agency in the nation, is responsible for ensuring the safety of the county’s 2 million children,^18^ emerged as an early partner in this work. With a new director at the helm, DCFS was replicating programs to leverage the neurobiology of trauma to inform public policy.^19^ The initiative, called Invest LA, sought to improve safety, wellbeing, and permanency for youth in part through investment in workforce excellence. Specifically, the platform called for each of the over 8000 employees of the department to understand the impact of toxic stress on brain development, promote child resilience, and apply their knowledge at the individual and system levels. As a result, DCFS partnered with DMH and UCLA to develop the first system-wide workforce development rollout.

### Training components

The training package is a two-pronged leadership capacity building effort that leverages both didactic (phase I) and reflective practice (phase II) components. Phase I training was delivered by four UCLA-affiliated trainers, three of whom were psychologists and one a master’s level clinician, all with extensive experience in trauma, child welfare, and supervision/management. Phase II training was delivered by three of the same psychologists who were also experts in reflective practice facilitation.

For phase I, all available managers and supervisors, which included DCFS executive leadership, were trained on a trauma- and resilience-informed care curriculum (*Building a Trauma-Responsive and Resilience-Strengthening Child Welfare System*) during a 1-day retreat that focused on the impact and prevalence of childhood trauma in the context of child welfare practices. The SAMHSA’s concept of trauma and guidance for a trauma- and resilience-informed approach was used and integrated into the trainings: (a) the effects of trauma on child development, brain, and behavior; (b) the impacts on relationships across the lifespan; (c) approaches to develop trauma- and resilience-informed practices for children, families, and systems; (d) implementation strategies to build resilience in children, families, and the self; (e) translation of learning into trauma- and resilience-informed practice; and (f) ways to cultivate skills to support wellness in self, staff, and clients.^6^ Importantly, the content and case examples were strategically tailored to the unique experiences of leadership to apply a trauma- and resilience-informed lens in managing and supervising staff. For example, one case example detailed a supervisee-supervisor interpersonal challenge and asked participants to discuss the supervisee’s strengths, moving away from “what is wrong with the supervisee?” to “what has the supervisee been through?” and finally to discuss how this influences the client.

For phase II, the lead operations partner and psychologist trainer followed up with each DCFS office to engage leadership stakeholders in ongoing reflective leadership consultation (thereafter, Reflective Circles) centered on the implementation of these strategies with their teams in practice. Reflective Circles are rooted in reflective practice frameworks, anchored in a deliberate safe process that encourages learning through active attention to knowledge and beliefs, as well as active reflection about experiences in relation to the self and others.^20,21^ Concretely, the goal of Reflective Circles was to help DCFS managers and supervisors of social workers and direct service staff to further develop their supervisory practices and better support the professional development of supervisees while fostering safety and trust, acknowledging strengths, and facilitating wellbeing. Specific goals for Reflective Circles included (a) reflection and building upon the strategies identified at the trauma retreat to develop actionable plans; (b) deepening of knowledge gained at the trauma retreat to build a trauma-responsive and resilience-strengthening CWS by increasing office supports; (c) transforming daily supervision practice to best support the professional and developmental needs of supervisees and model fostering a safe environment; and (d) recognizing secondary traumatic stress and moral distress to implement skills and strategies on building system-level resilience. Each site was a Reflective Circle cohort and participants attended at least four sessions.

It is important to note that although not the focus of the present study, a subsequent ongoing effort was initiated to train all DCFS staff, including social workers and line staff on trauma- and resilience-informed foundations and practice to support this leadership-supported effort.

### Procedures and participants

#### Phase I retreat

This study was conducted in compliance with the Institutional Review Board at UCLA. Roughly 1200 supervisors, managers, and executive team members across the 19 DCFS field offices that support the DCFS workforce were required by the department leadership to attend the 1-day in-person retreat. The executive team at DCFS includes directors, chief deputy directors, senior deputy directors, and executive leadership. At the outset of each 1-day retreat, and again after the retreat, participants were provided the option of a paper/pencil or electronic link to complete the pre-post-training surveys; electronic survey output data were generated using Qualtrics Software. Participants were asked to answer a set of questions to create a self-identifying code that was linked to their post-training survey responses.

#### Phase II reflective circles

Each regional administrator and division chief at the respective DCFS site was responsible for inviting staff to participate in the Reflective Circles based on their leadership and management roles and responsibilities to facilitate development of trauma- and resilience-informed systems of care within their team. As such, we were unable to capture a response rate for Reflective Circle participation given the training teams’ minimal involvement in recruitment. Ten Reflective Circle groups occurred virtually due to COVID-19 pandemic restrictions beginning in March 2020, and only three sites completed the series in-person prior to the pandemic. There was no way to link retreat with reflective circle participants.

A total of 123 participants attended at least three of the four Reflective Circle sessions. Of the total participants, 89 completed a post-Reflective Circle survey, which is the sample analyzed for phase II qualitative data. Sites were required to complete a four-session series; however, several sites requested additional sessions for their sites. Of the 13 sites, six completed the four required sessions and seven completed up to seven sessions total. Each session was approximately 90 min in length. Most sites completed a monthly session over the course of 6 to 8 months. Identifiable participant information that linked participants to qualitative survey responses was not collected to honor DCFS’s request for participant anonymity and to foster a trusting relationship with DFCS partners.

### Measures

#### Phase I retreat

Pre-retreat survey**.** Participants completed a paper/pencil or electronic pre-survey at the start of their respective retreat designed to gather age, gender, race/ethnicity, the highest level of formal education, and job title. The survey also consisted of a 10-item knowledge check to evaluate initial knowledge of trauma-responsive and resilience-strengthening child welfare systems and practices (e.g., What part of the brain is less accessible when a child has to spend too much time in survival mode?). This served as a baseline indicator of knowledge for each participant.

Post-retreat survey. The post-survey was administered immediately following the retreat and included the same 10-item knowledge check questions to evaluate changes in knowledge from baseline. Participants were also asked to complete a series of questions intended to evaluate participant perception of the impact the retreat had on their attitudes, values, and practice pertinent to trauma- and resilience-informed care (e.g., “As a result of this training…I will utilize trauma-informed lens in my work/practice”; “…I will plan to implement strategies to create a trauma- and resilience-informed system in my workplace”). Items were rated on a 4-point Likert scale ranging from 1 “Strongly Disagree” to 4 “Strongly Agree.” Participants were also asked to describe two strategies they plan to implement immediately to become more trauma- and resilience-informed in their work. Finally, participants were asked a few questions about the quality of the retreat intended to evaluate their satisfaction with the overall retreat experience.

#### Phase II Reflective Circles

Reflective Circles feedback form. Participants who attended the last Reflective Circle session were asked to complete an anonymous seven question electronic survey designed to capture their experience as part of the reflective consultation process. See Appendix for the list of questions.

### Data management and analytic approach

#### Quantitative data analysis

Quantitative survey data were collected from 1326 participants who completed a pre- or post-phase I quantitative survey. Data were initially retained for participants who filled out both pre-training and post-training surveys (*n* = 672), and subsequently removed cases for the following reasons: missing demographic data (gender, race, education, and job title, *n* = 154); job titles were not managerial or supervisory (*n* = 12); and outlier cases (*n* = 4 cases were retained for pre-training knowledge analyses but removed from post-training knowledge analyses). With the matched data (*n* = 672), we modeled the missing pattern in gender, race, education, and job title by regressing the missing indicator of each variable on the other variables and none of the missing indicators were significantly associated with the others. Therefore, we concluded that the missingness in our data is Missing Completely at Random, and it is reasonable to model only the complete cases. This resulted in 506 participants with pre-training data and 502 participants with post-training data. Table 1 shows demographic information for the final sample of participants. There are 398 females (78.7%); top education degrees are Master in Social Work (*n* = 248, 49%), BA/BS degree (*n* = 111, 21.9%), and MA/MS degree (*n* = 107, 21.1%); top racial groups are Hispanic/Latino (*n* = 230, 45.5%), African American/Black (*n* = 112, 22.1%), and White/Caucasian (*n* = 98, 19.4%); the majority of participants are supervising children’s social workers (SCSW, *n* = 402, 79.4%).

**Table 1:** Descriptive data on survey sample (*N* = 506)

Variable	*N* (%) or mean (SD), range
Female^1^	398 (78.7)
Race	
African American/Black	112 (22.1)
American Indian/Alaska Native	2 (0.4)
Asian/Pacific Islander	56 (11.1)
White/Caucasian	98 (19.4)
Hispanic/Latino	230 (45.5)
Other	8 (1.6)
Education	
BA/BS degree	111 (21.9)
BSW degree	29 (5.7)
MA/MS degree	107 (21.1)
MSW	248 (49.0)
PsyD	1 (0.2)
PhD	6 (1.2)
Other	4 (0.8)
Job title^2^	
RA/DC	10 (2.0)
ARA	29 (5.7)
SCSW	402 (79.4)
CSA/ASM	65 (12.8)
Knowledge scores	**Mean (range)**
Pre-survey	8.5 (2.4), 0–15
Post-survey	11.2 (2.5), 0–16
Improvement	2.6 (2.8)

1. 1.4% of the participants declined to state their gender. 2. RA/DC: regional admin/division chief; *ARA*, assistant regional administrator; *SCSW*, supervising children’s social worker; *CSA/ASM*, children’s services administrator/administrative service manager

Descriptive satisfaction data are reported, and two linear regression models were fit and analyzed to the pre- and post-training knowledge scores. Linear regression models were used to fit their associations with the demographic variables based on checking pre- and post-knowledge score normality through histogram plot graphs. The first model treated the pre-training knowledge score (pre-score) as the outcome and regressed on gender, race/ethnicity, education, and job title. The second modeled the post-training score (post-score) as the outcome and regressed on the pre-score and the same four demographic variables. The four categorical demographic variables—gender, race/ethnicity, education, and job title—were converted to binary dummy variables with the reference levels being female for gender, White/Caucasian for race/ethnicity, Master of Social Work for education, and supervising children’s social worker (SCSW) for job title.

#### Qualitative data analysis

Qualitative survey data were collected from 89 participants who completed the Reflective Circles feedback form comprising six open-ended questions. A “coding, consensus, and comparison” approach was used to analyze the participant responses.^22^ After reviewing a subset of responses, one senior coder developed an initial codebook containing 26 codes. This set of codes was used to independently code another subset of responses after which the coding team met to discuss refinements to the codebook. Two coders independently reviewed the 89 responses and met with the senior coder to reach agreement on any discrepancies in code application. The codes were further refined during this iterative process resulting in a final 27 codes. The authors (included coders) met to identify overarching themes that expanded upon the quantitative findings.

#### Integrating qualitative and quantitative analyses

Sequential QUAN/QuanQUAL mixed-method research designs were applied to the data wherein qualitative and quantitative data were sequentially collected and analyzed, beginning with quantitative data, for the primary purpose of confirmation and exploration (QuanQUAL and QUANqual, respectfully).^23^ Quantitative and qualitative findings were merged to identify convergent themes.

## Results

The aim of the study was to evaluate the feasibility and effectiveness of the training retreat and Reflective Circle sessions, specifically by assessing knowledge gain and practice change both quantitatively and qualitatively.

### Quantitative findings

The leadership training retreat (phase I) was feasible, with over 1200 leader-level individuals across all 19 DCFS offices trained. Data support a successful rollout with 95% of participants reporting overall favorable experiences with the training; 96% agree the training was coherent and well organized; 96% agree the trainer displayed mastery of the relevant issues and topics discussed; 96% agree the trainer was responsive to trainees; 96% agree the trainer helped trainees relate training content to practice; 95% agree the trainer’s teaching strategies were effective for me.

#### Knowledge improvement

Table 2 showed that in the post-score model, the pre-score was highly significant, and the other estimated effects were conditioned on the pre-score. On average, participants with 1-point higher pre-score resulted in 0.31 points (95% confidence interval = (0.23, 0.39)) higher post-score. Leaders with a BA/BS degree had a significantly lower post-score compared to those with a MSW degree (− 0.75, 95% CI − 1.22, − 0.28). Leaders in CSA/ASMs roles (i.e., administrative/managerial) had higher post-scores compared to those in SCSW roles (i.e., clinical supervisory) (0.9, 95% CI 0.33, 1.47). All the individual knowledge questions showed more improved correct answers than worsened incorrect answers in the post-survey compared to the pre-survey. All changes are significant by McNemar’s test for paired nominal data.

**Table 2:** Results of regression models on the knowledge scores

	Estimate	Std. error	*t*.value	Estimate	Std. error	*t*.value
(Intercept)	8.5	0.19	45^***^	8.9	0.39	23^***^
Pre-score	-	-	-	0.31	0.041	7.6^***^
Education^1^						
BA/BS	− 0.75	0.27	− 2.8^**^	− 0.75	0.24	− 3.1^**^
BSW	− 0.52	0.45	− 1.1	− 0.55	0.41	− 1.3
MA/MS	− 0.59	0.28	− 2.1^*^	0.065	0.25	0.26
Doctoral	0.049	0.9	0.055	− 0.83	0.81	− 1
Other	0.41	1.2	0.33	0.77	1.1	0.7
Male^2^	− 0.34	0.27	− 1.3	− 0.5	0.24	− 2.1
Race^3^						
Black	0.081	0.27	0.3	− 0.73	0.24	− 3^**^
Asian	− 0.32	0.35	− 0.92	− 0.2	0.32	− 0.64
White	1.4	0.29	4.8^***^	0.27	0.27	0.97
Other	1.2	0.79	1.5	-0.027	0.71	-0.038
Job title^4^						
CSA/ASM	0.65	0.32	2.1^*^	0.9	0.29	3.1^**^
ARA	0.34	0.45	0.75	− 0.3	0.41	− 0.75
RA/DC	− 0.82	0.75	− 1.1	0.7	0.68	1

Significance symbols: * *p* < 0.05; ** *p* < 0.01; *** *p* < 0.001. 1. MSW as the reference level; 2. female as the reference level; 3. Hispanic/Latino as the reference level; 4. SCSW as the reference level

#### Practice change

Most participants reported intention to change their practice with respect to trauma- and resilience-informed care. Specifically, 96.2% participants (47.8% strongly agree) plan to utilize a trauma-informed lens, 96.3% (38.1% strongly agree) know how to help their staff utilize a trauma-informed lens, 96% (42.7% strongly agree) will shift the perspective of themselves and their staff, and 96.4% (41.8% strongly agree) plan to implement strategies to create a trauma- and resilience-informed system.

### Qualitative findings

Participants were administered open-ended questions asking what they found most and least beneficial about the reflective circles. Important to note that although 89 surveys were completed, some respondents did not provide a response to all questions, so the total number of responses analyzed varies by question (range is *N* = 83–88). The following two themes emerged regarding the effectiveness of the Reflective Circles around its facilitation (open/safe environment, external facilitator, format, and scheduling) and reflection-grounded nature (team sharing/learning, trauma topics, and processing/reflecting). Participants also reflected on practice changes across their leadership and professional wellbeing. See Tables 3 and 4 for example quotes.

**Table 3:** Qualitative themes on the feasibility of reflective circles: representative quotes and respondent frequencies (*N* = 88)

Themes	Representative quote	Total *n (%)*
**1. Facilitation**		
*Open/safe environment (* +*)*	“An open forum with no real agenda to just express true feelings around strengths and barriers to move the work forward; honest dialogue.” “The level of comfort that was provided to allow team members to open up and process different issues that can have a negative impact in the workforce.”	49 (55.7)
*External facilitator (* +*)*	“Having a dedicated facilitator support and run these types of groups is necessary–specifically for our SCSWs and CSWs without management being present.” “…our facilitator, provided a safe and respectful 'space' to discuss our openness concerns, feelings and hopes for a more resilient…organization.”	10 (11.4)
*Scheduling ( −)*	“The impact of time. We start the communication that is important, but we have competing priorities that do not allow for the reflection one would hope to have in that space and time.” “The challenges we faced getting everyone on the team together at the same place and same time.”	18 (20.4)
*Format ( −)*	“Unfortunately, due to the pandemic the reflective circles were done via zoom. Having them face to face is more personal.” “It may be more effective if we can 'share' with those at the same level. It can be a bit intimidating to share concerns and issues in this type of setting with superiors around.”	17 (19.3)
**2. Reflection**		
*Team sharing/learning (* +*)*	“It was a great opportunity to talk to my co-managers about what is working well and what are some of the barriers that we are facing. It was great to hear how others are doing and gather tips from them.” “Hearing that we are all in this together and we have similar challenges at work and home.”	49 (55.7)
*Trauma topics (* +*)*	“Opportunity to discuss trauma and its effect on morale, performance, and self.” “Time set aside to reflect on a personal level rather than discussion of work-related concerns as in typical meetings. This has helped cohesiveness of team.”	21 (23.9)
*Processing/reflecting (* +*)*	“Taking the time to reflect – we are so busy and many of us wouldn’t take the time to do this had it not been a part of our meeting.” “Having the time and place to step back from the business of the day to process how the work impacts us, our coworkers, our clients.”	18 (20.5)

( +) Facilitator; ( −) barrier

**Table 4:** Qualitative themes on the effectiveness of reflective circles: representative quotes and respondent frequencies

Themes	Representative quote	Total *n (%)*
**1. Practice changes in professional wellbeing (*N* = 83)**		
*Self-growth*	“I am now able to look back at my practices with engaging staff and make improvements.” “Ability to see and address areas of growth needed, recognizing strength, and overall confidence.” “I believe I have opened up myself a bit more to self-improvement, especially during this time.”	22 (26.5)
*Awareness*	“I know that I need to continue to grow in the area of collaboration. I tend to look to fix problems, but this can ignore the benefits of coaching my team through the process of finding solutions” “Being more reflective and aware of my own issues and biases.” “I know that everyone deals with trauma or stress in different ways. Things that I may not really worry about may be a big worry for the other person.”	18 (21.7)
**2. Practice changes in leadership approaches (*N* = 84)**		
*Empathy/patience*	“Being reflective and open to identify what support others need. Allow for them to really have space to share what they are going through.” “Taking a different view of where someone is coming from (trauma experiences, etc.) in their responses and framing things through that lens.” “Validating this for us increases the collective empathy of the program for ourselves which transfers to our families.”	32 (38.1)
*Active listening*	“Listening to others more and the ability to praise my staff more.” “I believe that debriefing with colleagues has helped me navigate my own feelings in the tough times that presented while the reflective circles were held. I found myself closer to some of my colleagues and better able to understand their decision-making.” “I use more questioning/listening techniques rather than jumping to problem solving direction	24 (28.6)
*Openness and flexibility*	“I feel I am more open and reflective with my staff rather than just focusing on work and what is pending” “Sharing of struggles and more insight into strategies used to support staff during difficult times.” “I am more open and patient to allow staff to come up with their own solutions by gently guide them if needed.”	23 (27.4)
*Self-care*	“Do personal assessments and check in with staff regarding self-care” “Improved self-care as it relates to work demands. The pandemic has posed unique challenges in creating a balance between work and personal life. I am focused on improving in this area, so I don’t feel so burned out.” “I learned that I needed to slow down and to be more intentional about taking care of myself.”	10 (11.9)
*Trauma-informed culture*	“The interaction between colleagues seemed to open up or improve lines of communication.” “Discussions in unit meetings around how I want staff to take care of themselves and their family including taking a personal day when needed.” “More intentional about discussing impact of trauma with staff and exploring ways to cope.”	34 (40.5)
*Staff recognition*	“Being more intentional with acknowledging the good work and accomplishments of staff.” “I check in with my staff to ensure that they feel that their supervisor is listening and cares about help them solve issues that arise.” “Listening to others more and the ability to praise my staff more.”	33 (39.3)

#### Facilitation

Of the 88 participants, 67% underscored facets pertaining to facilitation as important to a successful Reflective Circle experience, namely having an open/safe environment and an external facilitator, while 40% discussed format and scheduling as top challenges.

Open/safe environment. Over half of the participants reported on the benefits of fostering an open and safe environment where participants could speak openly about their feelings, experiences, and perspectives; feel connected to others; as well as feel validated. Many participants mentioned benefitting from sharing their successes and challenges with others.

External facilitator*.* About 10% of participants mentioned the importance of having an external facilitator that could guide the Reflective Circles, chiefly emphasizing the importance of having a skilled facilitator who cultivated a safe space.

Scheduling*.* Twenty percent of participants reported that timing was challenging. Many participants indicated that it was challenging to schedule sessions amid competing priorities, with one participant stating that the demands of work made it difficult to maintain Reflective Circles.

Format*.* Nineteen percent of participants noted barriers with the format of Reflective Circles, specifically with the virtual format, driven by COVID-19 pandemic restrictions, and the group format of the sessions. Many reported on the challenges associated with having vulnerable conversations virtually with preference for in-person. Others expressed a desire to include only same-level staff, as opposed to various leadership levels in the same groups (e.g., executive management and clinical supervisor).

#### Reflection

Of 88 participants, over half highlighted some facet of reflection as key to a successful Reflective Circle experience, through team sharing/learning, discussions focused on trauma topics, and process/reflection prompts.

Team sharing/learning*.* Fifty-six percent of participants reported on the value of team sharing and learning. Participants appreciated the interactive nature of the sessions that allowed for effectively communication with team members. Some of the benefits reported were getting to know their colleagues better, feeling connected to their colleagues through shared commonalities, sharing resources, and exchanging strategies for addressing health and work issues.

Trauma topics*.* Twenty-four percent of participants mentioned that a focus on trauma topics was fundamental to the reflective group experience in that it fostered team cohesiveness, trust, and bonding. Discussing trauma topics with colleagues was important in creating an environment of support and understanding.

Processing/reflecting. Twenty percent of participants highlighted the benefit of processing and reflecting on work practices, leadership style, and trauma.

#### Practice change in professional wellbeing

Participants responded to an open-ended question regarding changes they noticed in their professional wellbeing practices and many respondents shared about self-growth and awareness.

Self-growth. Twenty-seven percent of participants reported experiencing self-growth because of Reflective Circles, predominately in interactions with their teams by employing different techniques, such as increasing support of staff needs and working more effectively as part of a team. A few participants became more mindful of their impact on others in the work setting, while others reported identifying areas of growth and making improvements.

Awareness. Twenty-two percent of participants reported an increased perceptiveness of both their own and staff challenges and strengths. Many expressed an increased awareness of the issues faced by staff, with one participant discussing their newfound approach of engaging staff with a lens of support and another acknowledging the importance of creating a safe space. A few participants shared their increased self-awareness in areas such as biases, professional wellbeing, and approaching challenges.

#### Practice change in leadership approach

Six themes emerged for practice changes in leadership, including empathy/patience, active listening, flexibility/openness, self-care, trauma-informed culture, and staff recognition.

Empathy and patience. Thirty-eight percent of participants reported increased empathy and patience with their teams. Some participants mentioned being more attentive to staff needs, while others noted adjusting their interactions with staff in response to staff needs.

Active listening. Twenty-nine percent of participants reported more use of active listening skills with staff, as evident by more frequent check-ins and other supportive interactions with staff.

Openness and flexibility. Twenty-seven percent of participants reported shifting to a trauma-focused leadership approach rooted in openness, transparency, and generally being more available to staff.

Self-care. Twelve percent of participants reported being more intentional about their own self-care, as well as actively promoting self-care activities among their staff.

Trauma-informed culture. Forty percent of participants reported more intentionality in implementing trauma- and resilience-informed strategies with their teams to foster a healthy work culture. Examples included implementing reflection activities and check-ins at the beginning of administrative meetings.

Staff recognition. Thirty-nine percent of participants described instituting staff recognition and praise for staff successes. Some highlighted reflecting with staff about their growth, needs, and struggles more intentionally.

## Discussion

This study is a key step towards understanding the feasibility and effectiveness of a large-scale trauma initiative geared towards leadership stakeholders within a CWS. Our findings suggest that a rollout of this magnitude is achievable, and like previous research,^7–9^ results showed this initiative was effective in improving knowledge and practice change in leaders. It was apparent that leaders with more education or those functioning in administrative roles demonstrated greater improvements. This is not surprising since administrators are farther removed from clinical work, and thus positioned for greater knowledge growth. It is also notable that over 90% of participants reported intentions to utilize and implement more trauma- and resilience-informed care strategies. It is evident that overall, a primary focus on leadership-level stakeholders was attainable and well received by the system at large, with several positive outcomes for leadership development.

Wide-scale training among leadership stakeholders is an important first step towards cultivating trauma- and resilience-informed strategies in a large CWS. The Reflective Circles were crucial to fostering their continued growth in trauma strategies. The reflective format allowed for a sense of safety in learning from each other and aided in their willingness to experiment with implementing strategies and sharing the outcome with their teams during subsequent sessions. Moreover, the use of an external facilitator ensured participants felt safe to reflect. This is aligned with the design of the groups, grounded in reflective supervision and consultation principles which promote continuous learning through reflection, collaboration, and regularity.^20,24^

Research suggests that active approaches like experiential, reflection, and collaborative learning are effective for leader development across sectors.^25,26^ Our study similarly showed personal and professional growth, including improved interactions with staff and implementation of trauma strategies. The ripple effects of leadership improvements in these areas are felt across organizations^15–17^, and therefore, investing in leaders who identify personally with the needed culture change is crucial. Results also support the two-stage process of first providing wide-scale knowledge and skill development, followed by reflective spaces to discuss the application of learned strategies. These findings showcase the importance of investing in both training types for leadership teams to reap the benefits of a positive organizational culture.

It is important to acknowledge several limitations of the study and highlight that more work in this area is warranted to better understand the specific mechanisms and core principles of the program that were most effective in the leadership-level changes reported. First, we used a self-report of intended practice change, and although behavioral intention often precedes behavior and can predict behavior change^13,15^, future research work should integrate objective measures of behavior change over time, such as collecting data from teams directly. Second, the inability to link the two data sets is a limitation to understanding distinctions across those who participated in Reflective Circles versus those who did not. The hope is that ongoing collaborations with our partners will provide future opportunities to gather linking data. Finally, it bears mentioning that our findings do not disentangle whether “external” and/or psychologically safe are the essential components of Reflective Circles. Future studies that characterize the implementation of this model in practice over time will be key to understanding what variations are essential. Despite the limitations, this is a promising model for engaging leadership-level stakeholders in trauma- and resilience-informed strategies.

## Implications for Behavioral Health

This study demonstrated that tailoring training for leadership that is rooted in trauma principles can improve knowledge and foster leadership characteristics and competencies that are consistent with a positive organizational culture. The positive impacts of trauma training and reflective spaces on leaders professional and personal growth hold promise for improving behavioral health systems broadly. This arguably has downstream impacts on all levels of a behavioral health ecosystem (individual, family, provider, and organization, and system) given its complexity. Most notably are the implications on health service delivery and clinical outcomes for children and their families. It is the hope that the present study can serve as a model for implementing trauma-informed practices at the leadership level for large behavioral health systems.

Understanding the impact of model variations is key to implementing in other areas of behavioral health. Additionally, the added benefit of providing similar day-long trauma-informed training to line staff and child social workers is not known at this time and will be the focus of future efforts with this project. It was also evident that in-person meetings were preferred by respondents over virtual, so future work needs to examine the impact of each platform on process outcomes like reflection and self-disclosure. Participants also mentioned the challenge of scheduling and time. Although this problem is not surprising, it is important to discuss with leaders at the planning stage of Reflective Circles to ensure all leaders have opportunities to participate. Finally, since the goal is to improve the outcomes of children and families in the child welfare system, evaluation of child and family-level outcomes are crucial. Both objective and subjective outcomes from family and child perspectives could elucidate on the impacts of leadership- and staff-geared trainings on the lives of children and their families.

## Appendix. Reflective circles feedback form

1. Would continuing reflective circles in your office be beneficial?YesNo
2. What supports do you need to sustain trauma- and resilience-informed care in your office?
3. What did you find most beneficial about the reflective circles?
4. What did you find least beneficial about the reflective circles?
5. As a result of the reflective circles, what shifts have you noticed in your leadership approach or skills?
6. As a result of the training and reflective circles, how have your own practices for professional wellbeing grown?
7. Any additional feedback is welcomed and appreciated.
